# Data-Derived Conceptual DFT Nucleophilicity Index

**DOI:** 10.1021/acs.jpca.6c00872

**Published:** 2026-06-29

**Authors:** Bartłomiej Fliszkiewicz, Hubert Suska, Stanisław Popiel

**Affiliations:** Faculty of Advanced Technologies and Chemistry 69698 Military University of Technology, Warsaw 00-908, Poland

## Abstract

The accurate characterization of nucleophilicity has attracted the attention of research groups worldwide for many years. So far, no universally applicable descriptor that correlates closely with experimental observations has been established. In this study, the behavior of the two conceptual DFT nucleophilicity indices is examined for a broad set of chemical compounds using semiempirical methods. The presented analysis reveals that the tested indices do not reliably capture experimental nucleophilicity trends when applied outside of small reference sets, leading to weak correlations with Mayr’s reactivity scale. To address these shortcomings, an empirical nucleophilicity index, *N*_*emp*_, is constructed through symbolic regression, incorporating frontier orbital energies, CDFT-based reactivity descriptors, atom-specific Fukui functions, local charge information, and solvent characteristics. Although the derived global model outperforms conventional descriptors (*R* ^2^ = 0.737), substantially higher accuracy (*R* ^2^ = 0.811) is obtained only when the models are further refined according to molecular scaffold. Collectively, these findings expose the inherent limitations of existing global CDFT nucleophilicity indices and demonstrate the potential of data-driven strategies to build context-aware reactivity descriptors.

## Graphical abstract

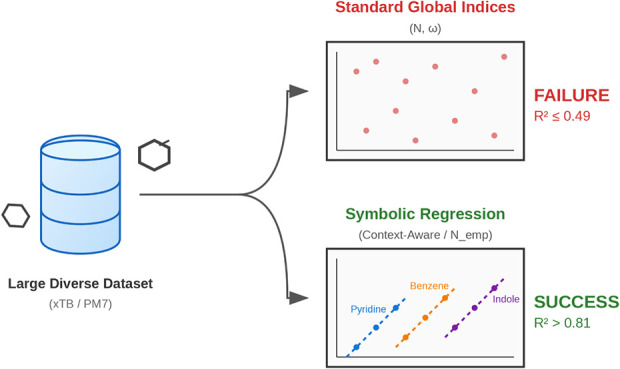

## 1 Introduction

Nucleophilicity refers to the propensity of a nucleophile to substitute a leaving group in a substitution process.^1^ A nucleophile is defined as an electron donor possessing a lone pair capable of forming a bond with a non-hydrogen atom.^2^ The earliest definitions of nucleophiles and electrophiles were proposed by Ingold in 1933.^3,4^ These fundamental definitions form the standard framework of organic chemistry around the world and continue to be used today.

Various concepts of conceptual/chemical density functional theory (CDFT) nucleophilicity indices have been proposed. In 2001, Chattaraj et al.^5^ studied the behavior of the inverse of the Parr’s et al.^6^ electrophilicity index as a new nucleophilicity index.

1 $$N=\frac{1}{\omega }$$

$$\omega =\frac{\mu^{2}}{2\eta }$$

In the above equation, μ and η denote the chemical potential and the chemical hardness of the compound, respectively.

An alternative conceptualization of nucleophilicity was proposed by Domingo et al.^7,8^ The authors introduced an N index, defined with respect to tetracyanoethylene (TCE).

2 $$N_{\left(D\right)}=\varepsilon_{HOMO_{\left(Nu\right)}}\left(eV\right)-\varepsilon_{HOMO_{\left(TCE\right)}}\left(eV\right)$$

An additional effort to define a quantitative nucleophilicity index was undertaken in 2011. At that time, Pratihar et al.^9^ applied the concept proposed by Chattaraj et al. and sought to establish a nucleophilicity ranking for 69 arenes and heteroarenes.

3 $$N=\frac{10}{\omega^{‐}}$$

The ω^–^ is electrodonating power parameter and could be defined as^10^

$$\omega^{-}=\frac{I^{2}}{2\left(I-A\right)},\mathrm{or},\omega^{-}=\frac{{\left(3I+A\right)}^{2}}{16\left(I-A\right)}$$

In 2024, Hoffmann et al.^11^ reported an *R* ^2^ of 0.78 between Mayr’s database nucleophilicity and an *N*_*G*_ index introduced in the study. Although the resulting *R* ^2^ was the highest among other nucleophilicity indices, the *N*_*G*_ index was calculated at the B3LYP/aug-cc-pVTZ level of theory for only 14 compounds.

4 $$N_{G}=-\left(\omega \left(1+\frac{\mu \gamma }{3\eta^{2}}-\frac{4\eta^{2}}{\mu \gamma }+\frac{4\eta^{4}}{3\mu^{2}\gamma^{2}}\right)\right)$$

$$\begin{array}{ll} \mu & =\frac{\varepsilon_{LUMO}+\varepsilon_{HOMO}}{2},,\eta =\varepsilon_{LUMO}-\varepsilon_{HOMO},\\ \gamma & =\varepsilon_{LUMO}-2\varepsilon_{HOMO}+\varepsilon_{HOMO-1} \end{array}$$

Although recent efforts have focused on the development of global CDFT-based nucleophilicity indices derived from frontier orbital energies, alternative strategies based on explicit nucleophile–electrophile interaction have also been reported. In this context Orlandi et al.^12^ proposed a multivariate linear regression approach to predict Mayr’s nucleophilicity parameters using reaction based descriptors obtained from DFT calculations. Their methodology relies on protonation as a universal probe reaction, allowing consistent extraction of electronic, steric, and solvation parameters from both the free nucleophile and its protonated form. The resulting model, constructed for 341 nucleophile-solvent combinations, achieved high predictive accuracy.

Despite ongoing research on proper description of nucleophilicity, there is still a gap that needs to be filled. Most nucleophilicity descriptors proposed in the literature show a high affinity for relatively small experimental data sets, which limits their application to only selected groups of chemical compounds, and applying them to structurally different compounds may result in an erroneous approximation of nucleophilicity.

This work is an exploratory investigation that (1) evaluates existing CDFT indices on large data sets and (2) shows that data-driven methods can reveal nucleophilicity patterns that theory-based descriptors fail to capture. Although the resulting empirical relationships still need to be validated on prospective data sets, they serve as a proof of concept that integrating local and global electronic descriptors with a structural context can substantially enhance the prediction of nucleophilicity. In order to reduce the calculation time, semiempirical quantum chemistry packages xTB^13^ and MOPAC^14^ are employed.

## 2 Materials and Methods

### 2.1 Mayr’s Database

The Mayr Database of Reactivity Parameters was exported from the official Database 2.0 Web site. It provides a collection of reactivity parameters, with particular emphasis on the nucleophilicity parameter N, which is the central focus of the study. The primary difficulty encountered was the lack of a complete set of SMILES strings for all listed compounds. Although common names for some substances were provided, many could not be identified because they were not present in publicly available resources such as PubChem.^15^ These missing chemicals were excluded from further analysis. The data set has also been restricted to the following solvents: water, tetrahydrofuran (THF), acetonitrile, dimethyl sulfoxide (DMSO), dimethylformamide (DMF), and dichloromethane because of the largest data range for these solvents. In Mayr’s Database nucleophilicity is calculated according to the following equation:^16^

5 $$\log k_{20{}^{\circ}C}=s_{N}\left(N+E\right)$$

In the above equation *E* is the electrophilicity parameter, *N* the nucleophilicity parameter (solvent dependent) and *s*_*N*_ the nucleophile-specific sensitivity parameter (solvent dependent).

The salts present in the database were turned into their dissociated states by removing the cation with RDKit.^17^ The database contained duplicates, which were results of both the salt removal process and the fact that some molecules contained two nucleophilicity centers with different values of the N parameter. In both cases, the duplicated rows that had the same SMILES and solvent values were removed, retaining the rows with the highest nucleophilicity parameter.

Molecules with nucleophilicity parameters outside 1.5 × *IQR* of the reference distribution were excluded, defining the applicability domain of the model. This reflects a deliberate scoping of the training set to the central range of nucleophilicity space rather than any deficiency in the reference data. This process reduced the database by 7 molecules. The final database consisted of 781 molecules. The removed molecules are shown in the Supporting Information in Figure S3.

In the article, the nucleophilicity values from Mayr’s database are denoted as *N*_*M*_.

### 2.2 Workflow

The geometries of the molecules were then generated with the ETKDG^18^ method from RDKit and saved as a mol file. The spin multiplicity of the molecules was calculated from the modulo 2 of number of molecule’s valence electrons. xTB calculations^13^ were conducted with the opt flag set as extreme, acc 0.01 and the analytical linearized Poisson–Boltzmann solvation model.^19^ The method used for the calculations was GFN1.^20^ The optimized molecule’s geometry saved in the .mol file was converted to the MOPAC^14,21^ input file with OpenBabel^22^ the MOPAC calculations keywords were PM7 PRECISE XYZ ENPART STATIC SUPER BONDS LARGE PRTXYZ DISP OPT ALLVEC EPS = <solvent’s dielectric constant> <multiplicity> CHARGE = <molecule’s charge> GRAPHF.

Based on the results, two nucleophilicity indices were calculated with eqs 2 (*N*_*D*_) and 4 (*N*_*G*_). In the study, the value of ε_*HOMO*_ of TCE of −9.061 eV was adopted, as calculated and reported by Hoffmann et al.^11^

Although both xTB and MOPAC were used for comparison of the correlation of *N*_*D*_ and *N*_*G*_ with *N*_*M*_, the latter part of the research—the equation deriving from the data was limited only to xTB because it has some implemented CDFT capabilities.

The results were gathered with Bash and Python scripts^23^ and then analyzed using Jupyter Notebooks,^24^ Python pandas,^25,26^ numpy,^27^ matplotlib,^28^ seaborn,^29^ scipy,^30^ scikit-learn^31^ and morfeus^32^ modules. Most of the data handling was conducted with the Snakemake^33^ workflow management system.

To assess whether the use of semiempirical calculations affects the accuracy of the models relative to DFT, a subset of DFT calculations were conducted. PySCF^34–36^ was used at the PBE0 ^37^/ma-def2-SVP^38^ level for both geometry optimization and single-point calculations, with implicit solvation described by ddCOSMO (water, ϵ = 78.36). Since PySCF does not provide CM5 ^39^ charges natively, Hirshfeld charges were computed using a dedicated Python package^40^ and subsequently converted to CM5 charges.^41^ Fukui *f* ^–^ and *f* ^+^ indices were obtained using the finite difference approach as the difference in CM5 charges between the states of N – 1, N and N + 1 electrons.

### 2.3 Symbolic Regression

Symbolic regression is a supervised machine learning task in which one searches the space of simple analytic expressions to generate accurate and interpretable models. The modeling results in an empirical expression that creates a relationship between the data and the target (experimental) variable.

To obtain the most accurate assessment of nucleophilicity, the modeling incorporated data for only a specific fragment of the molecule, since nucleophilicity is a localized property. In every molecule, an atom was chosen as the nucleophilicity center as described below. The atom is further referred to as the N atom. In the research, symbolic regression modeling was conducted with the following variables: μ, η, ω, γ, ε_*HOMO*_, ε_*LUMO*_, ε_*HOMO*–1_, CM5 charge, *f* ^–^ and *f* ^+^ of N atom, CM5 charges and *f* ^–^ of the N atom’s neighbors, and solvent accessible surface area, SASA, of the N atom. Data associated with the solvent were represented as μ_*solvent*_, η_*solvent*_, γ_*solvent*_, ω_*solvent*_, the dielectric constant of the solvent, ϵ_*solvent*_, the dipole moment μ_*D*_ and the solvent’s $\varepsilon_{HOMO_{solvent}}$ , $\varepsilon_{LUMO_{solvent}}$ and $\varepsilon_{HOMO-1_{solvent}}$ . The *f* ^–^ and *f* ^+^ are Fukui indices. Fukui indices are CDFT atom-based measures associated with reactivity toward an electrophile (*f* ^–^) and nucleophile (*f* ^+^). The center of nucleophilicity was chosen based on two steps:

1. The nucleophilicity sites were chosen according to the SMARTS rules in the same way as N. Ree et al.^42^ did and then passed to the second step. If no site was detected, then all atoms were passed to the second step.
2. Taking the assumption that the most nucleophilic site should have a maximum positive value of *f* ^–^ and a negative charge of CM5, the atom exhibiting the minimum value of a product of *f* ^–^ and the CM5 charge was designated as the N atom. This classification was applied because in some cases the maximum value of *f* ^–^ was attributed to a hydrogen atom.

The neighbors data was sorted in a similar way as N. Ree et al.^42^ sorted their data:

1. the neighbors were first sorted decreasingly based on their atomic number
2. in case of ties, the sorting was done decreasingly based on the sums of atomic numbers of neighbors’ neighbors, excluding the N atom
3. in case of ties, the sorting was done increasingly based on the neighbors’ CM5 charges.

The symbolic regression was conducted with the PySR^43^ package which is an implementation of a multipopulation evolutionary algorithm that searches over a broad, user-defined functional space without imposing a fixed equation form. The search space comprised the binary operators B={+,−,×,÷} and the unary operators $U=\left\{\exp\left(x\right),\;\log\left(x\right),\;\sqrt{x},\;x^{2},\;x^{3},\;x^{-1}\right\}$ , which produced a combinatorially large hypothesis space. Division, logarithm and exponential were additionally penalized with higher complexity costs to discourage unnecessary use. The maximum expression complexity, parsimony coefficient, and number of selected features were adjusted according to dataset size: for subsets with fewer than 30 points, the maximum complexity was reduced to 12 nodes, parsimony increased to 0.01, and feature selection limited to 4; for subsets between 30 and 60 points, these were set to 18 nodes, 0.005 and 6 features, respectively; for larger subsets the full settings of 30 nodes, 0.0032 parsimony, and 8 features were applied. The feature selection was applied via a gradient-boosting tree importance ranking, which is implemented in PySR package. The algorithm minimized a mean squared error loss over 1000 iterations across 20 independent populations of 100 individuals each, with numerical constants refined at each step via the BFGS algorithm with 3 random restarts. To balance predictive accuracy against interpretability, an adaptive parsimony scaling factor of 20 was applied, penalizing more complex expressions and naturally driving the search toward simpler functional forms. A fresh PySRRegressor instance was initialized for each solvent and scaffold subset to ensure reproducibility. The full code implementation is provided in the Supporting Information.

From the full Pareto front returned by PySR, candidate expressions were first filtered to a maximum complexity of 20 nodes and then ranked by the coefficient of determination *R* ^2^ evaluated on the training set. With the exception of the model trained on the water-solved molecular data, the top-ranked expression was adopted as the final model. The resulting models were assessed using the coefficient of determination *R* ^2^ and the root mean squared error (RMSE).

The symbolic regression was previously applied by Gonzalez et al. to model electrophilicity.^44^

## 3 Results and Discussion

### 3.1 *N_G_* and *N_D_*

The index *N*_*G*_ was calculated from the HOMO, LUMO and HOMO–1 energies calculated with both xTB and MOPAC. Table 1 shows the distributions of the index values. Although *N*_*G*_ calculated from MOPAC results have more reasonable values than xTB calculated (the range of values of more than 45k), in both cases the index does not correlate with the experimental values taken from Mayr’s database. As shown in Figure 1, both values of *R* ^2^ are close to 0 and the root mean squared error has a value >7. Although *N*_*D*_ appears to be more accurate, *R* ^2^ is at most 0.49 and RMSE > 5.

**Table 1:** Descriptive Statistics of Calculated *N_G_* and *N_D_* Values

	*N*_*G*_—xTB	*N*_*G*_—MOPAC	*N*_*D*_—xTB	*N*_*D*_—MOPAC
mean	–118.60	–19.01	–1.29	0.09
std	1692.78	2.53	0.91	0.72
min	–46964.1	–33.30	–4.00	–2.86
25%	–35.66	–20.22	–2.00	–0.35
50%	–28.49	–18.80	–1.44	0.11
75%	–25.24	–17.46	–0.67	0.61
max	392.06	13.60	2.54	1.84

**Figure 1:**
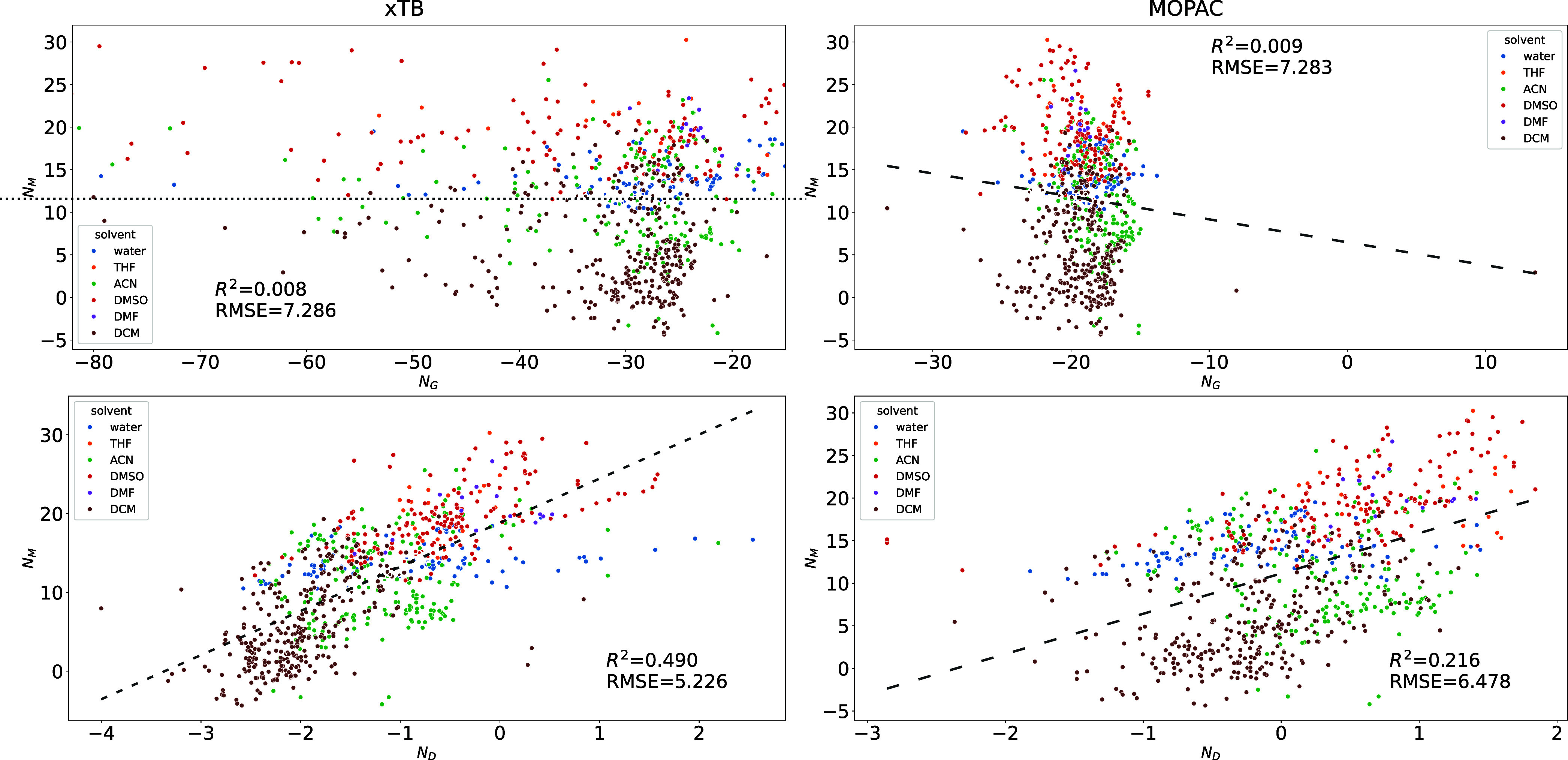
*N_G_* (upper plots) and *N_D_* (lower plots) calculated from MOPAC data (right) and xTB (left). The *x* axis of the upper left plot is limited from −82 to −15 (fifth and 95th percentiles).

The level of theory used in the investigation is less sophisticated than in the studies that introduced the *N*_*G*_ and *N*_*D*_ indices. Several (98) DFT calculations were conducted to eliminate the possibility that the root cause of the low correlation of *N*_*G*_ and *N*_*D*_ with *N*_*M*_ is the usage of semiempirical methods. Figure 2 shows the nucleophilicity indices calculated at the PBE0/ma-def2-svp theory level and their correspondence to the experimental values.

**Figure 2:**
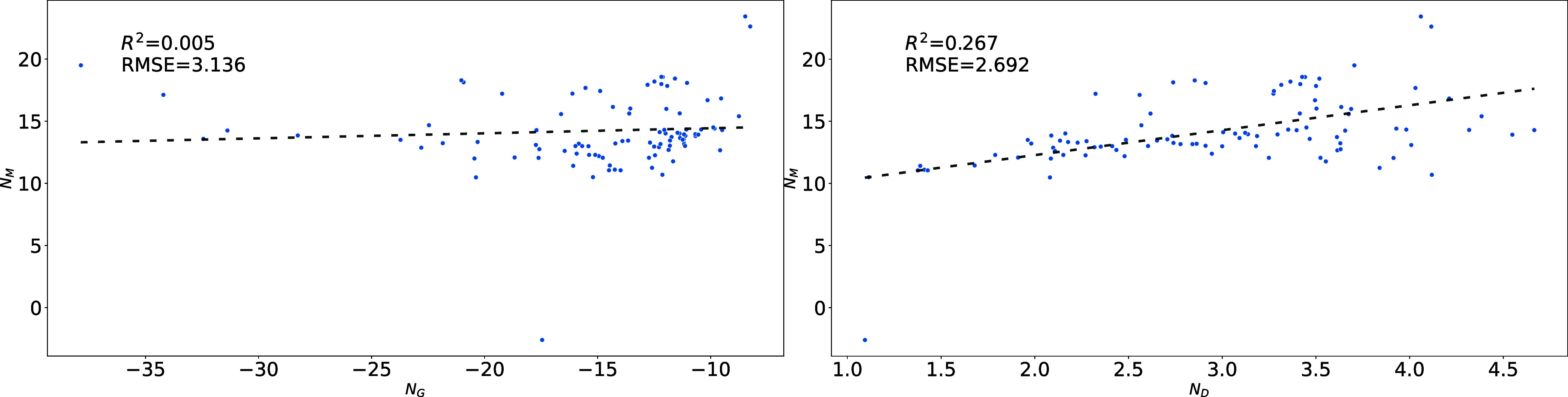
98 water solvated molecules’ nucleophilicity indices calculated with DFT level of theory. The molecules’ SMILES are listed in the Supporting Information.

Both indices show limited correspondence to the experimental values of nucleophilicity. The index *N*_*G*_ was tested with a limited number of compounds and the authors claim that “Further works on the prediction of experimental nucleophilicity should thus employ a larger dataset with advanced statistical tools.”

### 3.2 Symbolic Regression

#### 3.2.1 Whole Data Set

The *N*_*emp*_ derived from the complete data set used in the research is shown in eq 6. The symbolic regression identified the Fukui index of the N-atom, $f_{\mathrm{N‐atom}}^{-}$ , the HOMO energy of the molecule, the HOMO–1 energy of the solvent, $\varepsilon_{HOMO-1_{solv}}$ , the dipole moment of the solvent, μ_*D*_, the electronic chemical potential of the solvent, μ_*solv*_, and the CM5 charge of the N-atom, *q*_*N*–*atom*_, as the most relevant descriptors for nucleophilicity from the pool of candidate features. Although the equation accounts for the solvent, it does not consider steric hindrance of the chosen molecule’s nucleophilicity center.

6 $$N_{emp}=\mu_{D}+{\left(q_{N‐atom}-\mu_{D}f_{N‐atom}^{-}+2.89\right)}^{2}\left(\varepsilon_{HOMO}+\varepsilon_{HOMO-1_{solv}}+\mu_{solv}+31.0\right)+14.5$$

The *R* ^2^ of the global model is 0.737 and the RMSE is 3.766. The scatterplot of *N*_*M*_(*N*_*emp*_) is shown in Figure 3.

**Figure 3:**
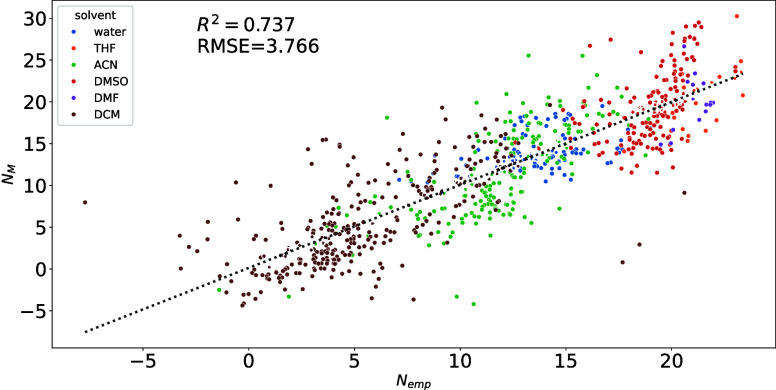
The symbolic regression modeling results. The black dashed line shows the linear regression of *N_M_* (*N_emp_*), and the points correspond to modeled vs predicted values of each compound. The data is also represented by the solvent from the Mayr’s database corresponding to each compound.

Although calculations were conducted with an implicit solvation method, the results show that the performance varies when the nucleophilicity of compounds is compared in the same solution. This phenomenon is probably due to the fact that the regression was dominated by data related to compounds solved in dichloromethane and acetonitrile, because they are the most numerous in the data set.

The interchangeability of semiempirical and DFT-calculated values in eq 6 was tested using descriptors computed for 98 water-solvated molecules. The model achieves *R* ^2^ = 0.089 and *RMSE* = 3 when evaluated on DFT-derived inputs, as shown in Figure 4. The limited transferability is expected, since the equation was derived from semiempirical data and the two methods differ in their parameterization and underlying approximations.

**Figure 4:**
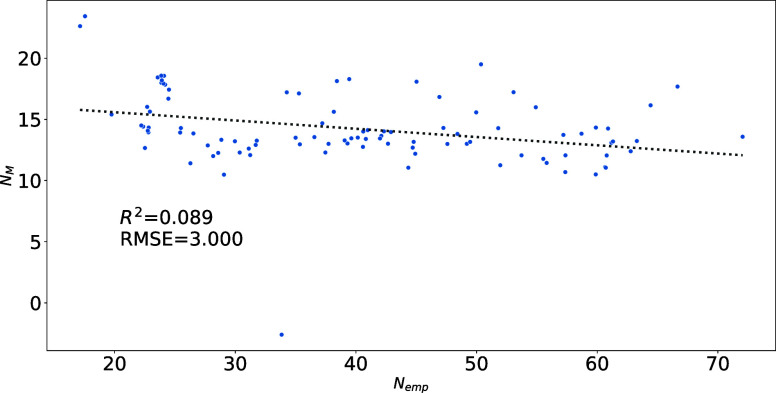
The results of applying global *N_emp_* model to DFT-calculated data.

#### 3.2.2 Equations Derived from Subgroups

In order to gain more mechanistic insights and possibly improve the predictive power of the empirical nucleophilicity index, a symbolic regression was conducted with the data grouped by solvent and by Bemis-Murcko scaffolds.^45^

Figure 5 shows scatterplots of symbolic regression results obtained with data grouped by solvent. The per-solvent equations show performance ranging from *R* ^2^ = 0.52 to *R* ^2^ = 0.78. Only the model fitted on the DMF data set achieves a higher *R* ^2^ than the global model, though this should be interpreted with caution given the small size of that subset (*n* = 15). Despite lower coefficients of determination, the per-solvent models yield lower RMSE values than the global model, which is expected since each model operates over a narrower range of target values. The substitution of xTB semiempirical descriptors with DFT descriptors in the water model was also tested; the resulting performance was *R* ^2^ = 0.056 and RMSE = 3.053, indicating that the two sets of descriptors are not interchangeable for this model. The corresponding scatterplot is shown in Supporting Information Figure S4A.

**Figure 5:**
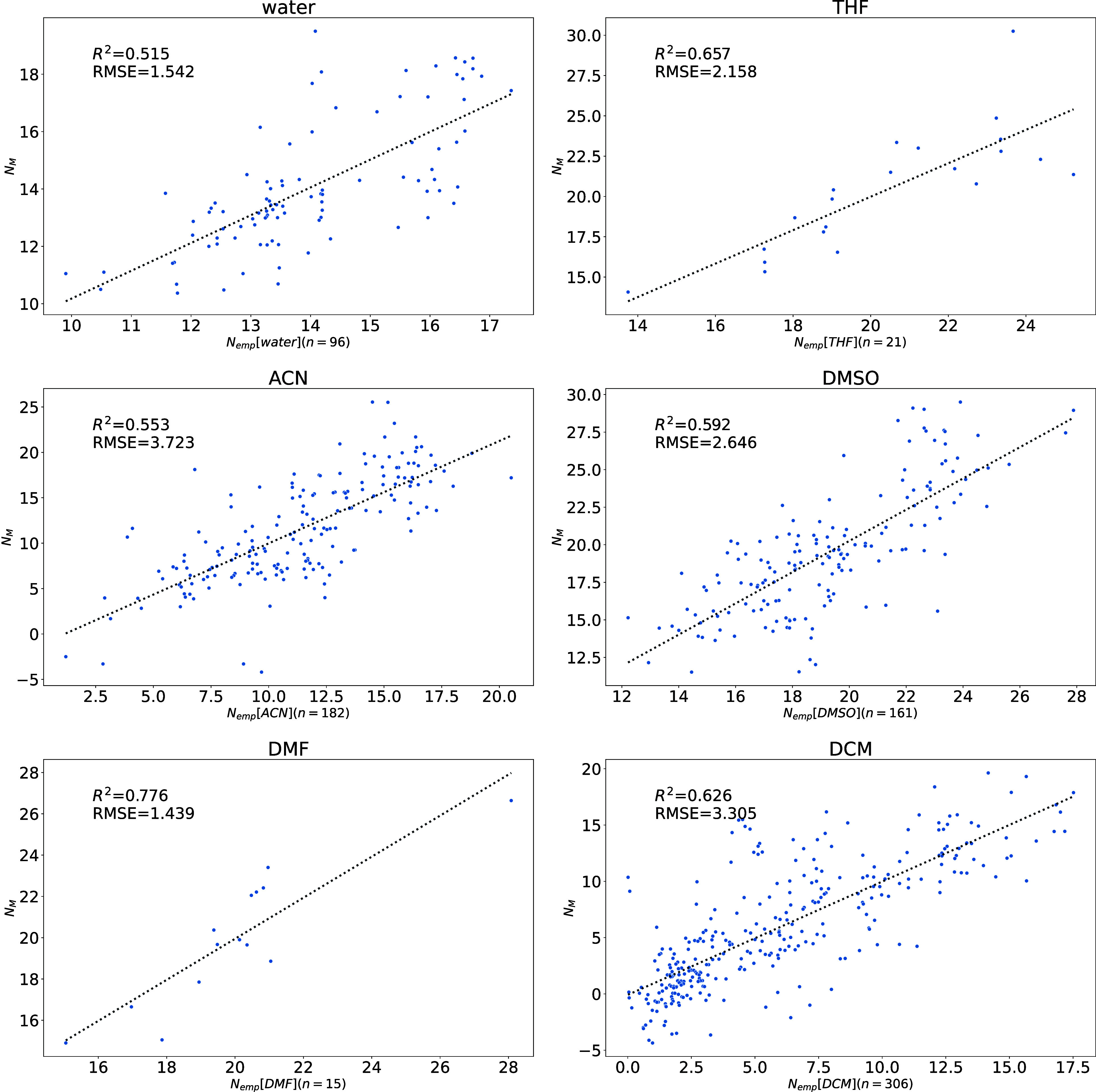
Per solvent symbolic regression. The *N_emp_* equations can be found in Supporting Information, Section S2A. The values in the parentheses are the number of compounds in the subset.

When Bemis–Murcko scaffold grouping was applied, the per-scaffold models achieved improved agreement with experimental nucleophilicity values. With one exception, the *R* ^2^ between *N*_*emp*_ and *N*_*M*_ is at least 0.75 across all scaffold groups. The lowest correlation, *R* ^2^ = 0.621, is obtained for the pyrrolidine scaffold (C1CCNC1), which is also the second smallest subset (*n* = 17). In particular, the equation derived from the benzene-scaffold subset (S8, *R* ^2^ = 0.888) accurately describes the nucleophilicity across water, acetonitrile, DMSO, DMF and dichloromethane (Figure 6), suggesting that the scaffold imposes the dominant functional form of the nucleophilicity relationship. The mean *R* ^2^ for all six scaffold models is 0.79.

**Figure 6:**
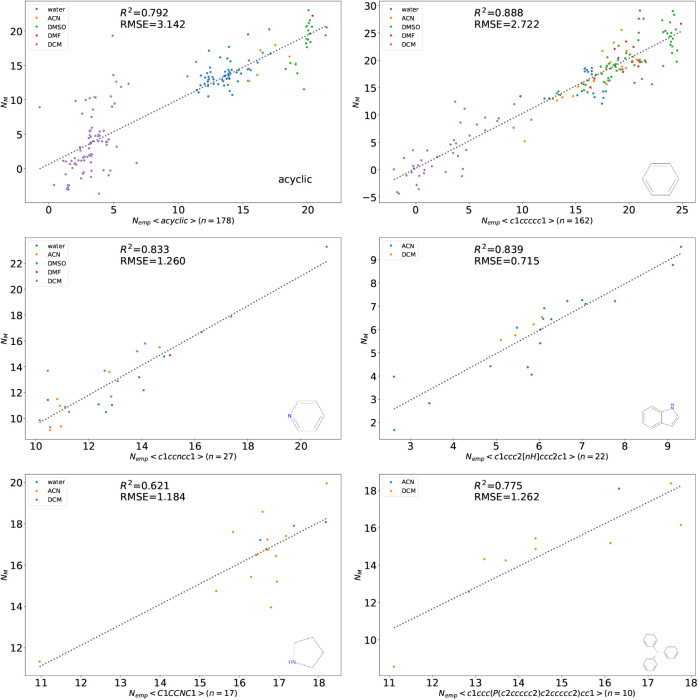
Scaffold based grouping nucleophilicity. In case no scaffold was detected, the compounds were classified as acyclic—upper left plot.

The improved predictive performance of the scaffold-grouped models arises from the fitting of the equations to compounds that share a common reaction mechanism. This suggests that nucleophilicity is more strongly dependent on the reaction mechanism than solvent effects, since solvent-based grouping yielded lower regression metrics. Another observation from the resulting equations is that, unlike the global equation (eq 6), most scaffold-specific models do not require explicit solvent descriptors—with the exception of the acyclic and benzene scaffold equations (S7 and S8). This suggests that implicit solvation may already capture the dominant solvent effect within each scaffold class and that additional solvent parameters are treated as redundant by the regression.

By combining the scaffold-specialized models with the global model—using scaffold-based equations for molecules whose scaffold was among the top six and the global equation for all remaining molecules—a combined model was constructed. Figure 7 shows the resulting scatterplot. Compared to the global model alone, the combined model improves *R* ^2^ from 0.737 to 0.811 and reduces RMSE from 3.766 to 3.194 across the entire data set of 781 molecules. As observed for the other models, the substitution of semiempirical descriptors with DFT-calculated values leads to a substantial deterioration in predictive performance; the combined model yields *R* ^2^ = 0.017 and *RMSE* = 3.117 (Figure S4B), indicating that the equation is not transferable between theoretical levels.

**Figure 7:**
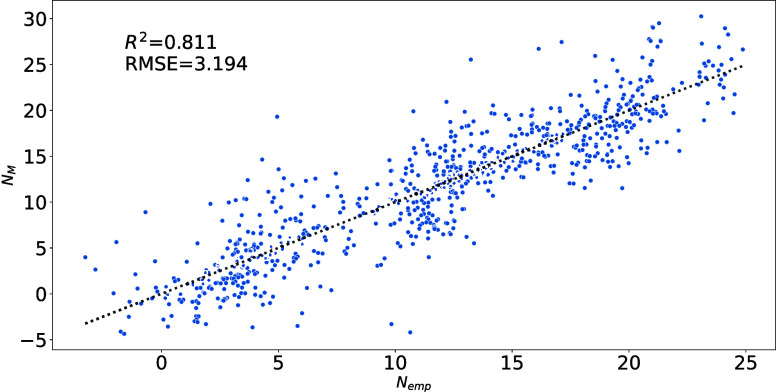
Scatterplot of *N_emp_* versus *N_M_* for the combined model (*R* ^2^ = 0.811, RMSE = 3.194, *n* = 781). Scaffold-based equations (S7–S12) were applied to molecules whose Bemis–Murcko scaffold matched one of the six scaffold groups; the global equation was used for all remaining molecules.

### 3.3 Context-Dependent Nature of Nucleophilicity

Table 2 summarizes the occurrences of descriptors in the per-solvent and per-scaffold *N*_*emp*_ equations. Among the global CDFT descriptors, ε_*HOMO*_ appears most frequently (7 occurrences), which is consistent with frontier molecular orbital theory: the highest occupied molecular orbital is directly associated with reactivity toward electrophilic reagents. The frequent occurrence of local descriptors—particularly $f_{\mathrm{N‐atom}}^{-}$ and *q*_*neighbor*1_ (5 occurrences each)—indicates that nucleophilicity is strongly dependent on the electronic properties of the nucleophilic atom and its immediate chemical environment. This presents a practical challenge for automated workflows, in which the nucleophilicity center must be identified without prior human involvement.

**Table 2:**
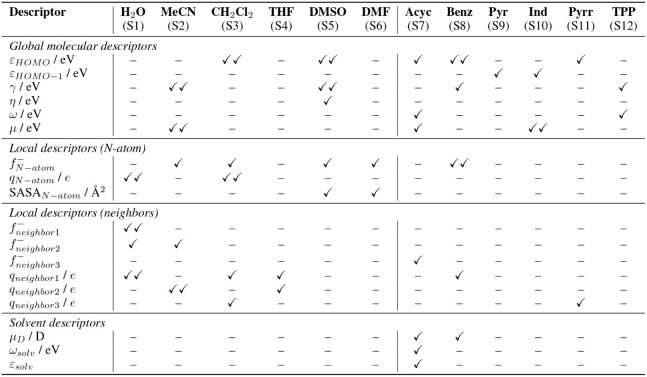
Descriptor Occurrence in Per-Solvent (S1–S6) and Per-Scaffold (S7–S12) *N_emp_* Equations^a^,^b^

a Legend: 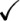 = appears once; 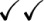 = appears multiple times; – = does not appear.

b Abbreviations: MeCN = acetonitrile, Acyc = acyclic, Benz = benzene (c1ccccc1), Pyr = pyridine (c1ccncc1), Ind = indole (c1ccc2­[nH]­ccc2c1), Pyrr = pyrrolidine (C1CCNC1), TPP = triphenylphosphine.

Interestingly, $f_{N‐atom}^{-}$ appears predominantly in the per-solvent equations and only one of the scaffold-based equations (benzene, S8), although it being commonly associated with nucleophilic reactivity. This may reflect the fact that the Fukui index is an intramolecular reactivity descriptor—it identifies the most reactive site within a given molecule rather than ranking reactivity between molecules. As such, it is less suitable to describe trends in intermolecular nucleophilicity, and descriptors that carry more absolute electronic information, such as *q*_*neighbor*1_ and ε_*HOMO*_, are preferentially selected by symbolic regression in a scaffold-grouped setting.

The appearance of hyperhardness γ in several equations warrants a comment. Although γ is formally a third-order energy derivative, it can be interpreted as a generalization of the chemical hardness η: when ε_*HOMO*–1_ = ε_*HOMO*_, γ is exactly reduced to η. In general, γ additionally encodes the energetic gap between HOMO and HOMO–1, providing information that η alone cannot capture. Its selection by symbolic regression suggests that the HOMO–HOMO–1 gap carries additional predictive information for nucleophilicity beyond what is encoded in the HOMO–LUMO gap alone.

Although several descriptors recur across equations, the variability in functional forms suggests that the appropriate description of nucleophilicity is chemical context dependent. It is also possible that the descriptor set used in this work does not encompass all variables that would constitute a complete model.

### 3.4 Comparison with Other Nucleophilicity Assessment Methods

Multiple computational strategies for predicting Mayr’s nucleophilicity parameter have been reported in the literature, and it is useful to contextualize the present model within this landscape. The combined model presented here achieved an *R* ^2^ of 0.811 and an RMSE of 3.194. Although these metrics are modest compared to some existing methods, the approach offers distinct practical advantages that make a direct comparison of accuracy alone misleading.

Methods based on probe reactions—namely methyl cation affinity (MCA) and the proton addition approach of Orlandi et al.^12^—achieve substantially better alignment with experimental values, with *R* ^2^ values of 0.89 ^46^ and 0.84 ^47^ (MCA) and 0.946 (proton addition). However, both methods require prior mechanistic knowledge: the user must know the exact regioselectivity of the reaction, and the reaction path modeling is computationally more expensive than characterizing a single compound in isolation. The present model relies solely on properties calculated for the nucleophile itself using a semiempirical method, which makes it well-suited for large-scale screening campaigns where reaction partners are not yet defined. It should also be noted that MCA correlates with the combined nucleophilicity parameter *N*_*M*_ *s*_*N*_ of Mayr’s equation (eq 5) rather than with *N*_*M*_ alone, and the correlation was established on 98 molecules spanning the full range of organic nucleophiles.^46^ Furthermore, the correlation was shown to deviate from linearity for weak nucleophiles and follow two distinct trends depending on whether the charge is delocalized or localized on the nucleophilic atom, which may limit its general applicability.

Liu et al.^48^ reported an *R* ^2^ of 0.92 and a *MAE* of 1.45 in a test set using an Extra Trees model with molecular descriptors—a method that shares the advantage of a single-reagent of the approach presented here. However, these results should be interpreted with caution. The reported implementation applies feature scaling to the entire data set prior to the train-test split and cross-validation, meaning the global mean and variance of the test set influence the training features. This constitutes data leakage and likely leads to inflated performance metrics. A valid implementation would require fitting the scaler exclusively on training indices within each fold. Although the feature importance analysis of Liu et al. identifies physically meaningful descriptors—including charge and E-state indices rooted in electronic structure—the Extra Trees model does not yield an explicit functional relationship between descriptors and nucleophilicity. The symbolic regression approach presented here produces an interpretable mathematical expression that offers a clearer basis for mechanistic reasoning.

## 4 Conclusions

The widely used and recently proposed nucleophilicity CDFT indices (*N*_*D*_ and *N*_*G*_), while effective for small sets of structurally related compounds, fail to provide accurate predictions when applied to large, diverse data sets, achieving *R* ^2^ values of 0.49 and 0.008 respectively on Mayr’s database. This limitation demonstrates that nucleophilicity is too multidimensional to be fully captured by a single universal functional form based on global molecular descriptors alone.

This exploratory study addresses that limitation by applying symbolic regression to derive empirical nucleophilicity indices from semiempirical quantum chemical calculations. The derived expressions exhibit substantially improved agreement with experimental data: *R* ^2^ = 0.737 for the global model, *R* ^2^ = 0.811 for the combined model, and up to *R* ^2^ = 0.888 for scaffold-specific models. Notably, the diversity of optimized equations across different chemical contexts reveals that different structural environments depend on different physical descriptors, reinforcing the context-dependent nature of nucleophilicity.

An important advantage of the proposed methodology is that it does not rely on electrophiles or specific probe reactions, allowing nucleophilicity to be assessed solely from molecular structure and solvent characteristics. This structure-based approach is computationally efficient and highly suitable for large-scale virtual screening campaigns. Nonetheless, as exploratory work, these models require validation on prospective data sets.

The observed mechanistic diversity suggests that pursuing a single universal nucleophilicity index may be fundamentally misguided. The markedly better performance of scaffold-based grouping (mean *R* ^2^ ≈ 0.79) compared to solvent-based grouping (mean *R* ^2^ ≈ 0.62) indicates that molecular architecture is the dominant factor governing nucleophilicity, with solvent effects playing a secondary role—a conclusion further supported by the fact that most scaffold-specific equations do not require explicit solvent descriptors. This ordering carries practical consequences: scaffold-specific equations should be applied when the molecular scaffold is known, whereas the global equation is more appropriate for previously uncharacterized structures.

Conceptual DFT does not yet offer theory-derived measures that adequately reflect the context-dependent character of nucleophilicity. Rather than persisting in the pursuit of universal descriptors, future work should prioritize context-sensitive models that distinguish between different mechanistic regimes and clarify why specific electronic factors become rate-limiting within particular structural environments. The present work demonstrates that data-driven methodologies can uncover mechanistic insights that, in turn, may support the development of more sophisticated theoretical frameworks.

## Supplementary Material



## Data Availability

The code, input data, and workflow management system script (Snakemake) are available on GitHub (https://github.com/BartlomiejF/empirical_cdft_nucleophilicity). Zenodo (DOI: 10.5281/zenodo.18182596) additionally contains the results of semiempirical and DFT quantum chemistry calculations.

## Notes

PySR code implementation; N_emp_ equations; list of molecules outside 1.5 IQR N_M_; N_emp_ models applied to DFT data—scatterplots (PDF)
