# Supplementary material for: Data-Derived Conceptual DFT Nucleophilicity Index
Source: J Phys Chem A. 2026 Jun 29;130(27):5141–9. doi: 10.1021/acs.jpca.6c00872 (PMC13359371; doi:10.1021/acs.jpca.6c00872)
Supplement: Supplementary file 1 [file jp6c00872_si_001.pdf]

Supplementary Information For Data-derived Conceptual DFT Nucleophilicity Index

BARTŁOMIEJ FLISZKIEWICZ, HUBERT SUSKA, AND STANISŁAW POPIEL

Faculty of Advanced Technologies and Chemistry, Military University of Technology, Warsaw, 00-908 , Poland, e-mail: bartlomiej.fliszkiewicz@wat.edu.pl

1. PYSR PARAMETERS

```
if n_points < 30:
    maxsize = 12
    parsimony = 0.01
    select_k_features = 4
elif n_points < 60:
    maxsize = 18
    parsimony = 0.005
    select_k_features = 6
else:
    maxsize = 30
    parsimony = 0.0032
    select_k_features = 8

model = PySRRegressor(
    maxsize=maxsize,
    niterations=1000,
    binary_operators=["+", "-", "*", "/"],
    unary_operators=[
        "exp", "log", "sqrt", "square",
        "inv(x)=1/x",
        "cube(x)=x^3",
    ],
    extra_sympy_mappings={
        "inv":  lambda x: 1/x,
        "cube": lambda x: x**3,
    },
    elementwise_loss="loss(prediction,target)=(prediction-target)^2",
    model_selection="best",
    populations=20,
    population_size=100,
    parsimony=parsimony,
    complexity_of_operators={
        "/":    2,
        "log":  2,
        "exp":  2,
        "cube": 2,
    },
    ncycles_per_iteration=550,
    turbo=True,
    batching=True,
    batch_size=50,
```

```
    adaptive_parsimony_scaling=20.0,
    warmup_maxsize_by=0.0,
    weight_add_node=0.79,
    weight_insert_node=5.0,
    weight_delete_node=1.6,
    weight_simplify=0.01,
    weight_mutate_constant=0.048,
    weight_mutate_operator=0.47,
    weight_swap_operands=0.1,
    optimizer_algorithm="BFGS",
    optimizer_nrestarts=3,
    should_optimize_constants=True,
    deterministic=True,
    random_state=42,
    parallelism="serial",
    progress=True,
    select_k_features=select_k_features,
)
```

2. N_{emp} EQUATIONS

A. per solvent

water

$$-33.3f^{-}_{neighbor2} + 15.5 - \frac{1}{q_{neighbor1} + \frac{\left(q^{2}_{N-atom} - 0.248\right)^{3}}{\left(q_{neighbor1} + f^{-}_{neighbor1}\right)^{3}}} \tag{S1}$$

acetonitrile

$$\left(\mu^{2} - 31.9\right)\left(f^{-}_{N-atom} - f^{-}_{neighbor2} - 0.306\right) + \log\left(\left(q_{neighbor2} + \frac{1}{\gamma + 0.0853}\right)^{2}\right) + 21.0 \tag{S2}$$

dichloromethane

$$\left((\varepsilon_{HOMO} + 10.3)^{2} - 3.97\right)^{2}\left(-q_{N-atom} + f^{-}_{N-atom} - q_{neighbor3} + \sqrt{\left(q_{N-atom} + q_{neighbor1} + 0.260\right)^{2}}\right) \tag{S3}$$

THF

$$15.4 + \frac{1}{q_{neighbor1} - \sqrt{q_{neighbor2}} + 0.213} \tag{S4}$$

DMSO

$$\eta + \varepsilon_{HOMO} - \frac{278}{\gamma\left(-\sqrt{f^{-}_{N-atom}} - 0.365\right) + \varepsilon_{HOMO}} + \frac{1}{sasa\,(\gamma - 0.692)} \tag{S5}$$

DMF

$$\sqrt{sasa} + 26.6 - \frac{2.39}{f^{-}_{N-atom}} \tag{S6}$$

B. per scaffold

acyclic

$$\varepsilon_{HOMO} + \sqrt{\mu_{D}\left(\mu f^{-}_{neighbor3}\omega^{2}_{solv} + \epsilon_{solv} + 111\right) - \frac{1}{0.138\omega - 0.807}} \tag{S7}$$

benzene, c1ccccc1

$$-\gamma + \left(-\varepsilon_{HOMO} - 2.53 - \frac{1.61}{\mu_{D} + q_{neighbor1} - 1.71}\right)\left(2f^{-}_{N-atom} + \varepsilon_{HOMO} + 12.4\right) \tag{S8}$$

pyridine, c1ccncc1

$$\sqrt{(7.49\varepsilon_{HOMO-1} + 87.8)^{2}} + 10.0 \tag{S9}$$

indole, c1ccc2[nH]ccc2c1

$$33.2 - 0.0684\left(\varepsilon_{HOMO-1} + \mu\right)^{2} \tag{S10}$$

pyrrolidine, C1CCNC1

$$-\sqrt{q_{neighbor3}} + 18.7 - \frac{1}{\varepsilon_{HOMO} + 11.6} \tag{S11}$$

TPP, triphenyl phosphine, c1ccc(P(c2ccccc2)c2ccccc2)cc1

$$3.98\gamma + 3.98\sqrt{(\omega - 12.8)^{2}} \tag{S12}$$

3. MOLECULES OUTSIDE 1.5IQR N_M

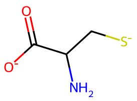

N_M = 23.43
Solvent: water

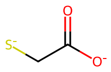

N_M = 22.62
Solvent: water

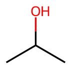

N_M = -2.6
Solvent: water

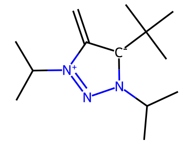

N_M = 31.92
Solvent: THF

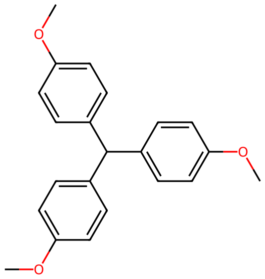

N_M = -7.0
Solvent: acetonitrile

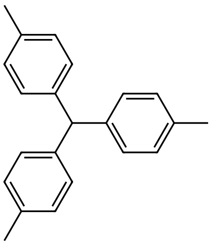

N_M = -8.8
Solvent: acetonitrile

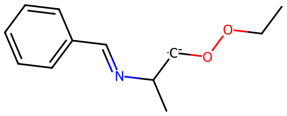

N_M = 30.82
Solvent: DMSO

4. N_{emp} MODELS APPLIED TO DFT DATA

A. Water - solved molecular model

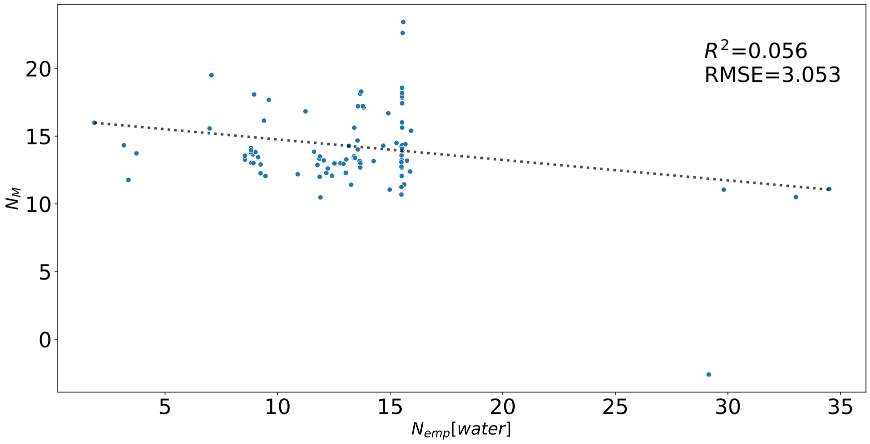

B. Combined model

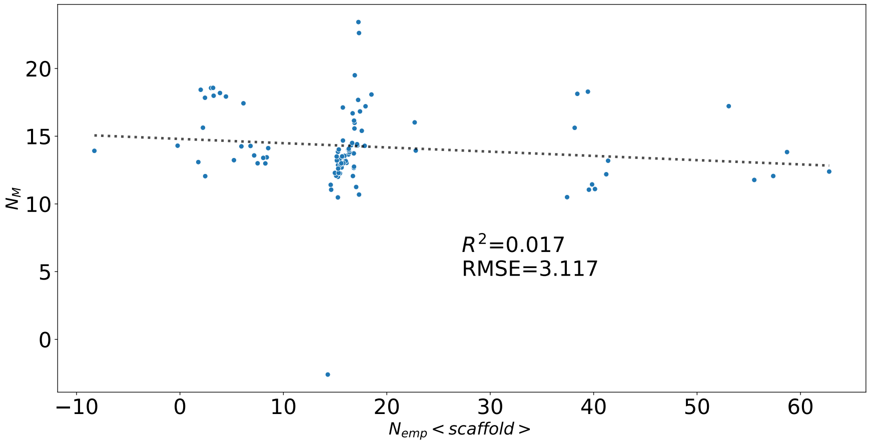

In case of detection of a scaffold that was not present in the training data, the global model was applied.
